# *Notes from the Field:* High Prevalence of Fentanyl Detected by the Maryland Emergency Department Drug Surveillance System — Baltimore, Maryland, 2019

**DOI:** 10.15585/mmwr.mm6923a3

**Published:** 2020-06-12

**Authors:** Zachary Dezman, Bradford Schwartz, Amy Billing, Ebonie Massey, E. Erin Artigiani, Julie Factor, Eric D. Wish

**Affiliations:** ^1^Department of Emergency Medicine, University of Maryland School of Medicine, Baltimore, Maryland; ^2^Center for Substance Abuse Research, University of Maryland, College Park, Maryland.

The toxicology screens of many hospitals include tests for common substances of abuse, including amphetamines, barbiturates, benzodiazepines, cocaine, cannabis, phencyclidine, and opiates. These tests, often enzyme-linked immunosorbent assays (ELISAs), might be limited by cross-reactivity and false-positives and false-negatives, and might only detect a specific set of substances. In 2018, a multicenter study of Baltimore-area emergency departments (EDs) showed a decline in the percentage of intoxicated patients with positive test results for opiates. At the same time, opioid-involved overdose deaths were increasing in Baltimore (*1*), suggesting that another opioid, not heroin, was the cause (*2*). Liquid chromatography-tandem mass spectrometry (LC-MS/MS) can be used to analyze urine specimens and identify a much wider variety of substances to which a person might be exposed (*3*). Unfortunately, LC-MS/MS is difficult to implement for point-of-care testing, and it would be cost-prohibitive to test every patient. The Maryland Emergency Department Drug Surveillance (EDDS) system institutes limited LC-MS/MS testing when there are changes in patient signs and symptoms that are not explained by routine testing, suggesting that a new substance is being used. This report documents the frequent identification of fentanyl among ED patients suffering from overdoses in Baltimore, which would not have been possible without the assistance of EDDS.

Since 2016, EDDS has obtained quarterly exports of deidentified encounter data and routine urine drug screen results for patients with an *International Classification of Diseases, Tenth Revision* (ICD-10) encounter code of T40 (poisoning by, adverse effect of and underdosing of narcotics and psychodysleptics [hallucinogens]), or if one or more of the following main complaint reason codes are included: drug overdose (378), overdose, accidental (807), overdose intentional (808), HPI-toxidrome, or overdose, ingestion (301056). The EDDS sites include seven academic and community EDs located in Baltimore City and Prince George’s County, Maryland.*

Previous pilot studies using LC-MS/MS conducted at University of Maryland, Midtown Campus (MTC), one of the EDDS hospitals, suggested an increasing prevalence of fentanyl among patients evaluated for drug overdoses. In 2016, 28% (19 of 69) of patients evaluated at the MTC ED with complaints of overdose and synthetic cannabinoid use had positive test results for fentanyl and fentanyl metabolites (*3*). During the 2017 Memorial Day weekend (May 27–29), four of eight patients treated in the MTC ED with complaints of overdose or intoxication had positive test results for fentanyl and related metabolites (*4*). A subsequent study of patients evaluated in the MTC ED with complaints of overdose or withdrawal or seeking substance use disorder treatment was conducted during February–April 2018. On-site fentanyl testing by urine rapid chromatographic immunoassay (Rapid Response, BTNX, Inc.) found that 83% of 76 patients had used fentanyl, whereas only 25% of these patients had positive test results for opiates using the hospital’s opiate screen (*5*). These findings suggested that fentanyl alone, not in combination with heroin, was being used more frequently and would otherwise be undetected among patients. In late January 2019, MTC and the University of Maryland Medical Center (UMMC) initiated routine fentanyl testing for all patients who undergo urine drug testing using the Vitros 5600 Immunoanalyzer with fentanyl immunoassay reagents (ARK Diagnostics^†^).

Fentanyl test results were available for 408 of 441 patients with specimens submitted to EDDS by UMMC and MTC for January–December 2019. Seventy-two (18%) of the 408 patients had only an ICD-10 T40 encounter code, 236 (58%) had one or more of the listed complaint codes, and 100 (24%) had both. The MTC and UMMC results were combined because there were no substantial differences between sites in patient mean age (47.6 years versus 47.7 years), proportion male (66.9% versus 68.3%), or proportion who reported nonwhite race (78.1% versus 74.1%), respectively. During January–December 2019, 83% (340 of 408) of patients had positive test results for fentanyl, making fentanyl the most commonly detected drug during 2019. Among the 340 patients with positive test results for fentanyl, 70% were male, 81% reported nonwhite race, and the median patient age was 50 years. Consistent with previous UMCC findings, fentanyl was the most prevalent drug, detected in 73% (45 of 62) to 87% (125 of 143) of patients tested in each of the four calendar quarters in 2019 (Figure). The opiate screen was negative for 55% (186 of 340) of the fentanyl-positive specimens. Among all fentanyl-positive specimens, 44 (13%) were positive for fentanyl alone. Most patients with positive test results for fentanyl were exposed to multiple substances: 61% (208 of 340) of specimens contained two or more drugs or drug classes in addition to fentanyl.

**FIGURE:**
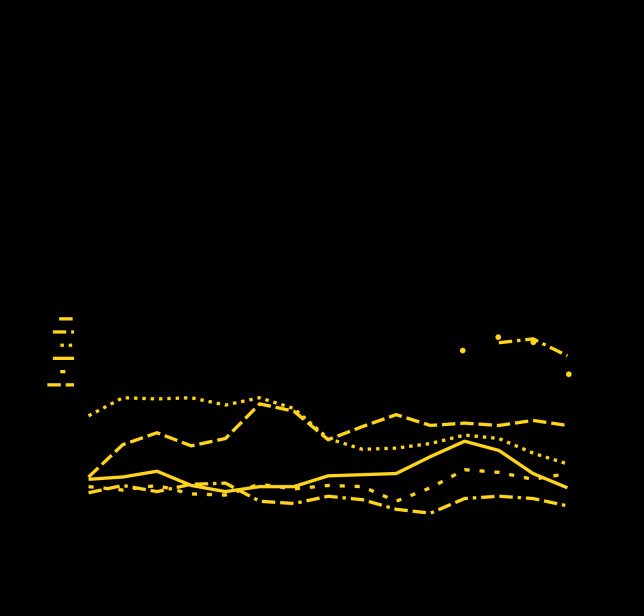
Percentage of substances detected in emergency department (ED) patients evaluated for drug overdoses*^,†^ (N = 1,707) — University of Maryland Medical Centers,^§^ January 2016–December 2019 * Lines indicate 2-quarter moving average. Amphetamines, barbiturates, and phenylcyclohexyl piperidine (PCP) results not shown because of low occurrence. ^†^ Numbers for benzodiazepines and methadone vary slightly because not all specimens were tested for all drugs each period. **^§^** University of Maryland Medical Center and Midtown Campus combined.

The high frequency of fentanyl use found in the population, especially in those patients who tested negative for opiates, demonstrates that regular fentanyl testing addressed a gap in patient care. A hybrid approach of rapid testing for the most common substances combined with limited LC-MS/MS testing to detect emerging substances enabled researchers and hospital systems to respond to the latest trends in substance use affecting patients. Programs like EDDS, which rely on robust institutional partnerships, are a model for other areas of the country seeking to address their own changing patterns of substances use in their community. The high prevalence of fentanyl detected in this study only applies to patients in Baltimore, and the findings might not be generalized to other cities or hospitals. Immunoassays validated for fentanyl might not detect all of the clinically relevant fentanyl analogs. Hospitals should consider conducting validation studies with analytical methods such as LC-MS/MS to determine what substances are being used in their communities.

## Footnotes

* University of Maryland: Midtown Campus; University of Maryland Medical Center; Baltimore-Washington Medical Center; University of Maryland: Saint Joseph’s Medical Center; Prince George’s County Medical Center; University of Maryland Laurel Medical Center; and University of Maryland Bowie Health Center.

† https://www.ark-tdm.com/products/urine-drug-tests/fentanyl/pdfs/ARK_Fentanyl_Assay_Rev04_June_2018.pdf.
